# Mouse Model of Cytomegalovirus Disease and Immunotherapy in the Immunocompromised Host: Predictions for Medical Translation that Survived the “Test of Time”

**DOI:** 10.3390/v10120693

**Published:** 2018-12-06

**Authors:** Matthias J. Reddehase, Niels A. W. Lemmermann

**Affiliations:** Institute for Virology, University Medical Center and Center for Immunotherapy of the Johannes Gutenberg-University Mainz, Obere Zahlbacher Str. 67, D-55131 Mainz, Germany

**Keywords:** adoptive cell transfer, CD8 T cells, cytomegalovirus, hematopoietic cell transplantation (HCT), hematopoietic reconstitution, humanized mice, immune control, immune evasion, immunotherapy, interstitial pneumonia, mouse model, T lymphocytes, viral pathogenesis

## Abstract

Human Cytomegalovirus (hCMV), which is the prototype member of the β-subfamily of the herpesvirus family, is a pathogen of high clinical relevance in recipients of hematopoietic cell transplantation (HCT). hCMV causes multiple-organ disease and interstitial pneumonia in particular upon infection during the immunocompromised period before hematopoietic reconstitution restores antiviral immunity. Clinical investigation of pathomechanisms and of strategies for an immune intervention aimed at restoring antiviral immunity earlier than by hematopoietic reconstitution are limited in patients to observational studies mainly because of ethical issues including the imperative medical indication for chemotherapy with antivirals. Aimed experimental studies into mechanisms, thus, require animal models that match the human disease as close as possible. Any model for hCMV disease is, however, constrained by the strict host-species specificity of CMVs that prevents the study of hCMV in any animal model including non-human primates. During eons of co-speciation, CMVs each have evolved a set of “private genes” in adaptation to their specific mammalian host including genes that have no homolog in the CMV virus species of any other host species. With a focus on the mouse model of CD8 T cell-based immunotherapy of CMV disease after experimental HCT and infection with murine CMV (mCMV), we review data in support of the phenomenon of “biological convergence” in virus-host adaptation. This includes shared fundamental principles of immune control and immune evasion, which allows us to at least make reasoned predictions from the animal model as an experimental “proof of concept.” The aim of a model primarily is to define questions to be addressed by clinical investigation for verification, falsification, or modification and the results can then give feedback to refine the experimental model for research from “bedside to bench”.

## 1. Introduction

Human cytomegalovirus (hCMV) is the prototype member of the *β*-*Herpesvirinae*, which is a subfamily of the family *Herpesviridae* (for an overview of CMV taxonomy, see Reference [1]). Medical interest in hCMV is based on its highly pathogenic potential in the immunocompromised host or, upon congenital infection, in immunologically immature fetuses, which result in multiple-organ disease and birth defects known as the cytomegalic inclusion disease (CID), respectively (for overviews, see References [2,3,4]). Reactivation of latent hCMV from the transplant or from recipients’ organs in consequence of the therapy of the primary disease is a medical challenge at all transplantation centers worldwide. Clinical examples are hemato-ablation in the case of hematopoietic malignancies followed by hematopoietic cell transplantation (HCT) and graft-versus-host disease (GvHD) prophylaxis or an immunosuppressive prophylaxis for preventing graft rejection in the case of solid organ transplantation (SOT).

CMV virus species exist in essentially all mammalian host species and have co-speciated with their respective host in eons of co-evolution, which results in an intricate virus-host adaptation reflected on the viral side by sets of “private genes” not shared between different CMV species [1,5] and resulting in a strict host-species specificity of CMVs [6,7,8]. As an inevitable consequence, no animal model can be expected to precisely reflect human infection in all aspects. Any conclusion from any animal model must, therefore, be seen with some caution regardless of how close to humans the chosen host species may be. However, non-human primates (NHPs) and their CMVs are considered to be models closer to the human disease than other animal models [9,10,11,12,13,14]. It is important in this context to note that CMVs of NHPs also critically differ from hCMV not only genetically but also phenotypically (for examples, see Reference [11]). The detection of unconventional, MHC class II (MHC-II) restricted CD8^+^ T cells in an NHP model of vaccination based on CMV vectors [14] awaits confirmation in humans.

As a further layer of complication, increasing evidence indicates substantial genetic and pathogenetic differences not only between recent clinical isolates of hCMV and commonly used laboratory strains such as AD169 and Towne, which are highly attenuated and restricted in cell-type tropism as a result of genomic deletions during long-term high-passage propagation in cell culture, but even among independent clinical isolates [15,16,17,18]. As emphasized by Wilkinson and colleagues [15], the problem of mutation in vitro is not restricted to large-scale genetic changes found in laboratory strains. Instead, mutations are also rapidly selected in low-passage strains. This means that any isolate expanded in cell culture for use in experiments likely differs from its archetype as which it was present in the patient from whom it was originally isolated. This led these authors to suggest to discredit the frequently used term clinical strain by plausibly arguing that all strains are clinical by origin but no longer clinical once propagated in cell culture [15]. Notably, work by the group of T.F. Kowalik revealed high genomic diversity of hCMV in humans, which suggests rapid intra-host evolution. hCMV genotypes isolated from different organs of an individual patient were found to be as divergent as genotypes sampled from different patients, which is a phenomenon referred to as “compartmentalization” [16,17,18]. Thus, if a chance to test hCMV in an animal model would exist, we would face the problem to decide which hCMV genotypes are most representative for predictions concerning hCMV disease in patients. Wilkinson and colleagues advocated the bacterial artificial chromosome (BAC)-derived strain Merlin since it *bona fide* represents the original virus isolate from which it was derived [15]. Nonetheless, also Merlin does not represent the inter-strain variance that shapes hCMV disease. Laboratory strains AD169 and Towne, which dominated research on hCMV for decades, do not qualify for predicting clinical hCMV pathogenesis. Early work comparing these strains with low-passage strain Toledo in SCID-hu mice with human thymus-liver implants revealed the strong attenuation of the laboratory strains for growth in human tissues and, thus, a missing viral histopathology [19].

Further in this line of argument, playing with the non-existing scenario that hCMV would replicate in an animal model, such a heterologous virus-host model would be highly artificial because the intricate virus-host adaptation by co-speciation would be disrupted. We, therefore, strongly advocate the view that, due to “biological convergence”, homologous models testing animal species’ CMVs in their respective host is closer to human infection in key parameters than testing of hCMV in a notional animal model of hCMV infection could ever be.

Interstrain variance likewise exists for murine CMV (mCMV) in wild-derived isolates (reviewed in Reference [5]). Mutation of the *MCK-2* gene, which has a dual function by encoding a viral chemokine homolog and a component of the entry receptor gH/gL/MCK-2 [20,21,22], prevents virus cell-associated dissemination to the salivary glands in immunocompetent mice [23,24,25] by a failure to hijack patrolling monocytes [26]. This excludes MCK-2 deficient mCMV as a model for host-to-host transmission via saliva-based mucosal infection. However, MCK-2 is not essential for viral histopathology and replication in the salivary glands in immunocompromised mice [27], which shows us that the intended model matters. Besides the notable exception of MCK-2, empirical knowledge indicates that mCMV is genetically and phenotypically more stable than hCMV and maintains virulence and the host cell-type range even after long-term propagation in a fibroblast cell culture. Specifically, the important issue of endotheliotropic vs. non-endotheliotropic strains of hCMV (reviewed in Reference [28]) is not a critical issue with mCMV. In the immunocompromised murine host, fibroblast-propagated mCMV, for instance, the prototypic Smith strain as well as BAC-derived viruses (reviewed in [29,30]) infect a plethora of different cell types in essentially all organs [31] including endothelial cells [32,33,34] and cause lethal multiple-organ CMV disease [31] closely resembling the broad cell-type tropism and histopathology that is characteristic of hCMV disease in immunocompromised patients [35]. Notably, the mCMV model opens the possibility to study the pathogenetic impact of interstrain variance with experimentally-designed variants, namely virus mutants generated by site-directed BAC mutagenesis and competing with each other in vivo upon experimental co-infection [36].

Despite the strong analogy in histopathology, results from mCMV research in general are not well accepted in clinical science. In this brief review, we focus on the specific mouse model of CMV immunotherapy in the immunocompromised HCT recipient in order to highlight predictions that have proven valid and translatable to hCMV disease and immune intervention in HCT patients.

## 2. The Importance of “Model Design” for Fitting a Clinical Correlate

The question of why the medical acceptance of studying mCMV infection is limited should bother all who work in this field. A key to understanding may be to think about the meaning of the term “model”. Studying mCMV infection is science in its own right and has contributed numerous basic insights into CMV-host interactions [37]. Some of these are likely specific for the particular virus-host adaptations while others revealed general principles such as principles of antigen processing and presentation that are valid despite the noted differences between mouse and human immunology [38]. In this sense, studies on mCMV infection of mice have already provided valuable ”paradigms” for virus-host interactions. It is our strong opinion, however, that the terms paradigm and model are not synonyms. Model implies that studies in the mouse serve to address a medical question that is of interest and need in humans. Therefore, if one plans to use mCMV as a model for hCMV, the first task is to define the medical question to be addressed. To become a valid model of predictive value for hCMV disease, key parameters must be adjusted to reproduce a clinical correlate as close as feasible instead of using experimental protocols that are way off clinical reality. Cornerstones defined by hCMV infection of humans are the following.

(i) Except in the case of congenital hCMV infection, transmission occurs under birth or at any time after birth, most frequently via mucosal exposure to virus-containing saliva in early childhood. This often happens in daycare centers or in more-child families where multiple contacts increase the probability of transmission. In immunocompetent humans, even in newborns, primary hCMV infection is associated only with unspecific feverish symptoms rarely-to-never diagnosed as hCMV infection. Primary infection can be associated with a mild mononucleosis, which is detected only by sheer chance in a routine hemogram. However, even then, hCMV is rarely taken into consideration as the causative agent. As an important consequence, the time of primary hCMV infection of an individual in the hCMV-seropositive but healthy part of the population is, in general, unknown. Likewise, the viral dose during the first encounter is an unknown parameter. Presumably, the initial dose is very low rather than high. This lack of information is a critical issue since the mouse model, by comparing infection of newborn versus adult mice, predicts that the time of primary infection and the viral dose determine the viral intra-host spread and the extent of amplification by viral replication. This defines the viral load/genome number present during viral latency after resolution of productive primary infection, which, in consequence, determines the probability for reactivation from latency to recurrent infection [39] and associated long-term effects on the immune system including “memory inflation” and immuno-senescence [40].
  Conclusion: Primary mCMV infection of immunocompetent mice with doses of infection or via routes of infection that result in severe morbidity or even lethality with organ manifestations disqualifies as a model for the natural biology of CMVs.
(ii) hCMV infection of immunocompromised HCT recipients in the period between hematoablative anti-leukemia therapy and immunological reconstitution by HCT develops into multiple-organ CMV disease with the lungs as a major manifestation site clinically presenting as interstitial pneumonia with an often lethal outcome if left untreated (reviewed in Reference [41]).
  Conclusion: mCMV strains of low virulence that fail to cause disease, including interstitial pneumonia, in immunocompromised mice disqualify for a model agent. As mentioned above, attenuated laboratory strains of hCMV would also fail in this respect in “humanized” mice with human tissue implants.
(iii) hCMV disease in humans implies that the individual has no genetic resistance to hCMV infection. If resistance by genetically-determined intrinsic defense mechanisms that prevent virus replication in host cells exist, the mechanisms would be of interest but would not apply to individuals who are at risk of becoming CMV patients.
  Conclusion: Only genetically-susceptible host genotypes such as strains of mice susceptible to mCMV infection qualify for use as a model for hCMV disease.
(iv) It is a hallmark of hCMV infection of humans that resolution of productive primary infection results in a state of “latency” that is defined by the absence of infectious virions despite maintained presence of viral genomes from which reactivation to recurrent productive infection can occur by triggering mechanisms that are still under investigation.
  Conclusion: A model system must reproduce resolution of productive infection, establishment of latency, and reactivation to productive recurrent infection.

## 3. The BALB/c Mouse Model of CMV Disease

In the early 1980s, the group of U.H. Koszinowski established the BALB/c mouse model of CMV disease and immune control. Certainly, other choices might have been as suitable, but, in retrospect, the BALB/c mouse model has proven valid in mimicking hCMV pathogenesis in humans with respect to the “cornerstones” discussed above.

(i) Intraplantar infection of young-adult BALB/c mice at an age of 8–10 weeks with moderate doses of the Smith strain of mCMV, namely 10^5^ PFU, led to a strong immune response in the draining regional lymph node [42]. Viral spread to vital host organs was very limited and, in accordance with the absence of detectable histopathology, morbidity was not observed and all mice survived long-term without any single exception [43]. Thus, in fulfillment of condition (i) defined by hCMV infection, mCMV is not pathogenic in the chosen immunocompetent model host.
(ii) When the very same route and dose of infection were used for BALB/c mice immunocompromised by a hemato-ablative, sub-lethal dose of total-body γ-irradiation prior to infection, which is a regimen used in the human correlate for leukemia therapy preceding HCT, the virus was spreading to essentially all organs. This caused an extensive viral histopathology with an invariably lethal outcome [43]. Importantly, the lungs were found to represent a major manifestation site of CMV disease in this model. Thus, in fulfillment of condition (ii), mCMV proved to be highly pathogenic and caused lethal disease with a histopathology and cell-type tropism resembling hCMV disease in humans, interstitial pneumonia included.
(iii) From the viewpoint of the host, the approach described for (ii) simultaneously also proved that the BALB/c strain of mice is not genetically resistant to mCMV infection by cell-intrinsic antiviral defense mechanisms. Thus, in fulfillment of condition (iii), BALB/c mice are susceptible to mCMV infection and, therefore, qualify as a model host. Besides BALB/c, the mouse inbred strain C57BL/6 is frequently used for studying mCMV infection since it has the advantage that most of the available knock-out, knock-in, and transgenic mice are based on the C57BL/6 genetic background (for an overview, see Reference [44]). A comparison between these two strains with respect to the course of mCMV infection has been comprehensively reviewed by the group of A.B. Hill [45]. C57BL/6 is considered to be a “resistant” strain, which raised the question if mCMV infection of C57BL/6 mice and C57BL/6-based mouse mutants has a clinical correlation that qualifies them as model hosts for studying CMV disease. Investigations into the mechanism of resistance identified Ly49H^+^ natural killer (NK) cells, which do not exist in BALB/c mice, as the cellular mediators of resistance that become activated via the virally-encoded ligand m157 [46,47,48,49]. This means that the resistance results from a mouse strain-specific branch of innate immunity and not from a cell-intrinsic antiviral defense mechanism. Accordingly, mCMV replicates and causes disease in C57BL/6 mice that are immunocompromised by hemato-ablative treatment [50] and the complication imposed by m157 ligation of the activatory NK cell receptor Ly49H can be avoided by infection with an mCMV mutant in which the m157 encoding gene is deleted [49]. Thus, with this in mind, C57BL/6 and mouse mutants derived thereof also qualify as model hosts, and other mouse strains may do as well.
(iv) Numerous studies early on demonstrated latent mCMV infection in immunocompetent mice and reactivation to recurrent infection by diverse immunosuppressive experimental regimes. For reviews of pioneering work on mCMV latency, see References [51,52]. For more recent reviews on mCMV latency and reactivation in HCT, kidney-SOT, and sepsis models, see References [53,54,55,56]. Importantly, in a study performed with mice infected as neonates, the lungs, which is a major organ site of hCMV disease after HCT [41], proved to be a predilection site of mCMV infection not only in the acute primary infection but also for viral latency and reactivation [57]. The same conclusion applied to a study on the establishment of mCMV latency in the lungs after experimental HCT [58]. As mentioned above, the time of primary infection is decisive in that infection of immunocompetent adult mice in comparison to neonatal mice results in limited viral spread to organs and limited viral replication within tissues. This accordingly results in a lower number of latent viral genomes harbored in tissues and, thus, also a lower incidence of reactivation to “recurrence” upon immunosuppressive treatment [39].
  Conclusion: The pathobiology of mCMV in the mouse model resembles its hCMV-human counterpart in critical parameters including the main organ site of disease manifestation even though virus-host co-speciation definitively must have generated distinct differences between the model host and human infection in details. In particular, one must keep in mind that “biological convergence” has created analogous functions for non-homologous genes and their gene products, which resulted in different molecular mechanisms. As a consequence, the mouse model can predict principles, but not, or at least rarely, the precise molecular mechanisms that are effective in hCMV infection.

## 4. Why Are Models Needed at All?

It is trivial to note that models serve to address questions that require scientific experiments under defined and controlled conditions for replacing statistical evidence obtained from observational clinical investigation with “proof of concept”. Critical conditions include:

(a) Genetic Host Parameters

Individual genetics is highly heterogeneous in cohorts of patients enrolled for clinical studies, which complicates the interpretation of results. Even more problematic than genetic variance as such is the fact that control cohorts never precisely reproduce the genetic variance represented in the test group. It is possible to stratify for age, gender, and numerous other host parameters, but it realistically is almost impossible to achieve a complete match between control and test cohorts in clinical trials. Although one can select patients for certain genetic parameters during enrollment, for instance the expression of a particular HLA antigen by HLA-typing in transplantation studies, one cannot experimentally manipulate host genetics.

(b) Host Infection Parameters

As mentioned above, the individual infection history is important for the extent and duration of acute primary infection, for the latent viral genome load, and for the risk of reactivation, but is unknown for a human patient or control volunteer in terms of time and viral dose. Quantification of the latent viral genome load in organs would require performing quantitative PCR on organ biopsies prone to sampling error, and collecting such data is illusionary especially for the control cohort. In addition, each individual harbors her/his “private” virus or even virus strain mixture based on co-infection, super-infection, and intra-host selection. Thus, it is almost impossible to achieve a complete match between the control and test cohorts in clinical trials. Like with host genetics, hCMV genetics cannot be experimentally manipulated by site-directed viral mutagenesis for an in vivo analysis. It is clear that hCMV mutants can be constructed for tests in cell culture, organoid culture, or “humanized” mice. However, unlike in animal models, testing viral mutants designed for identifying gene functions on an organismal level, in particular gene functions involved in virus-host interaction and host immunity, is ruled out in humans.

## 5. Why a Mouse Model?

When considering phylogenetic relatedness, the mouse model is the worst model with respect to both host species and virus species [1,5]. In addition, although more recent studies on comparative developmental anatomy of the murine and human placenta suggested more analogies than was previously thought [59], the differences between mice and humans in placental anatomy disfavored the mouse and favored the guinea pig as a model for congenital hCMV infection [60] in which diaplacental transmission of the virus at the fetal-maternal interface is most important [61]. However, as the brain of newborn mice corresponds in its developmental stage to that of human fetuses at the end of the second trimester, infection of newborn mice can serve as a model for CMV neural fetopathy such as CMV pathogenesis in the central nervous system after completion of organogenesis (for reviews, see References [62,63]). On the contrary, infection of newborn mice is not an appropriate model for brain involvement in CMV embryopathy after congenital infection occurring during organogenesis. Rat CMV, more for technical reasons, is a favored model for SOT and CMV-associated vascular diseases [64] even though the kidney-SOT mouse model also significantly contributed to our understanding of CMV reactivation triggering in SOT with the important message that ischemia/reperfusion injury can kickstart the viral transcriptional program [65]. As already discussed above, NHP models, and the rhesus macaque model in particular, are most closely related to the clinical situation in both virus and host parameters despite subtle but notable differences such as the existence of unique immunomodulatory genes not found in hCMV [11]. Therefore, each model has advantages and disadvantages and needs to be seen with some caution since only humans are humans and even NHP-CMVs co-speciated with their NHP host differ distinctively from hCMV.

Besides practical considerations including lower ethics concerns as well as affordable test and control group sizes for achieving statistical significance, which are limiting factors in NHP models, a scientific advantage of the mouse model is the easier manipulation of host genetics.

## 6. Are “Humanized” Mice the Future in CMV Research?

Our answer is “Yes, but…”. Mice “humanized” hematopoietically and with human tissue implants open the possibility to test in vivo hCMV replication in implanted human tissues and in human cells of hematopoietic cell lineages. This is of particular advantage for an in vivo evaluation of antiviral compounds, as virally-encoded as well as host-encoded enzymes involved in viral replication and gene expression are not absolutely conserved, and even homologous proteins involved in virus maturation are not identical. Nonetheless, caution is required when biological activity of an antiviral pro-drug requires host metabolism in tissues other than the implanted tissues and pharmacokinetics may differ between humans and implant carrier mice. Humanized mice allow us to test the control of viral infection in the implanted human tissues by tissue-infiltrating human hematopoiesis-derived cell types involved in innate and adaptive immunity such as effector cells and regulatory cells as well as antigen-presenting cells (APCs) including dendritic cells (DCs). Furthermore, approaches of cellular immunotherapy can be tested by adoptive transfer of human donor-derived lymphocytes. Contributions of humanized mouse models to understanding hCMV disease and immune control have been reviewed previously [66,67].

Nonetheless, this modern and overall promising approach also has its limitations to bear in mind. CMV disease, which is associated with host morbidity and lethality, results from the infection of multiple functional organs instead of implants in a ”mouse test tube”, so that protection against morbidity and lethality by therapeutic approaches can only be extrapolated from viral histopathology in the implanted tissues. More critically, virus spread in the host cannot be tested under conditions of a physiological anatomy, and the cross-talk between immune cells and stromal or parenchymal cells, except those in the tissue implants, is adulterated by a disturbed cytokine/chemokine network in which ligand-receptor pairs do not always cross-signal in both directions. We see a particular problem in the chimerism between human-derived lymphocytes and murine stroma in lymphoid tissues where T and B cells home, proliferate, and mature. Nonetheless, such models have their justification if appropriate questions are asked in the awareness of the limitations. Table 1 compares strengths and weaknesses of conventional and humanized mouse models, and the list most likely can be extended.

## 7. Immunotherapy of CMV Disease by Adoptive Immune Cell Transfer: An Early Prediction from the BALB/c Mouse Model that Went to Clinical Application

The BALB/c mouse model established in the 1980s, even though it is seemingly unsophisticated, has paved the way for clinical trials of preemptive immunotherapy of hCMV disease in HCT recipients by combining HCT with the adoptive transfer of virus-specific CD8^+^ T cells. Back to the roots: as described above, immunocompetent BALB/c mice survived a moderate-dose intraplantar mCMV infection, whereas immunocompromised mice all died of multiple-organ CMV disease under otherwise identical conditions. Onset of death was preceded by a fulminant infection of essentially all vital organs corresponding to massive tissue destruction by viral cytopathogenicity in the absence of immune cell tissue infiltrates, which excluded an immunopathology [43].

At that time, it was still open to question if viral replication in immunocompromised mice by a total-body γ-irradiation reflected the absence of antiviral immune control or, alternatively, reflected an altered permissiveness of host tissue cells due to genotoxic stress-associated repair responses or due to cytokine signaling based on a “cytokine storm” elicited by cell death of hematopoietic cells. An apparent difference between immunocompetent and hemato-ablated mice was presence and absence, respectively, of immunological priming in the regional lymph node draining the site of infection. Wereasoned that in case immune cells would determine antiviral control, cells isolated from the draining lymph node of infected immunocompetent donor mice should rescue infected immunocompromised recipient mice upon adoptive cell transfer by controlling the infection. This turned out to be the case. The responsible cell type was identified as the CD8^+^ T lymphocyte subset (then known as Lyt-2^+^) by the finding that depletion of CD8^+^ T cells from the transferred lymphocyte population abrogated antiviral control in the recipients despite the presence of CD4^+^ T cells (then known as L3T4^+^) and all non-T cells contained in the lymph node cell population. Accordingly, immune control remained after depletion of CD4^+^ T cells. CD8^+^ T cells were protective not only when transferred shortly before infection but also when transferred six days after infection when the virus had already colonized the lungs, even though more cells were needed for such an “immunotherapy” of an established tissue infection [43].

In the early experiments, the model was focused on events in designated HCT recipients without actually performing HCT. When combined with HCT, immunotherapy by adoptive CD8^+^ T cell transfer bridged the critical phase between infection and HCT-based endogenous lympho-hematopoietic reconstitution of antiviral CD8^+^ T cells. This was reflected by reduced lethality, faster clearance of productive infection, a reduced viral genome load in organs of survivors during latency, and a reduced risk of reactivation upon secondary immune cell depletion. Adoptive CD4^+^ T cell transfer failed to protect and infection of HCT recipients with no CD8^+^ T cell transfer was lethal when combined with the depletion of endogenously reconstituted CD8^+^ T cells. This demonstrated that reconstitution of antiviral CD8^+^ T cells is critical for surviving mCMV infection after experimental HCT [68].

In a number of follow-up publications over the years (for more recent reviews, see References [69,70,71]), it was shown that CD8^+^ T cells represent direct antiviral effector cells that need no cooperation from other cell types, which were still present in the CD4-depleted lymph node cell population transferred in the original experiments discussed above [43]. Purified memory CD8^+^ T cells derived from the spleens of latently infected mice also protected against CMV disease upon adoptive cell transfer [72]. Notably, purified memory CD4^+^ T cells alone failed to protect and their co-transfer up to a four-fold excess over memory CD8^+^ T cells did not improve protection. This indicated that protective memory CD8^+^ T cells neither require CD4^+^ T cell help nor profit from it for an immediate antiviral effector function upon adoptive cell transfer [72].

Not unexpectedly, the avidity of T cell receptor (TCR) binding to the complex formed by binding of an antigenic peptide to a presenting MHC class I molecule (pMHC-I complex) proved to be an important parameter for protection efficacy of transferred CD8^+^ T cells [71,73]. Strikingly, donor CD8^+^ T cell populations devoid of specificities for “immunodominant” epitopes, that is epitopes against which most of the natural response is directed, maintained protective efficacy. This indicated that non-immunodominant epitopes are as good as immunodominant epitopes in mediating protection. For an explanation, there is evidence to suggest that “immunodominance”, which is defined in quantitative rather than qualitative terms, results from high numbers of low-avidity cells that contribute little to protection [74,75,76]. Short-term CD8^+^ T cell lines (cytolytic T lymphocyte lines, CTLLs) mediated protection regardless of whether the CTLLs were poly-specific or monospecific and almost any viral epitope mediated protection regardless of its physiological “immunodominance” in the donor (reviewed in Reference [71]).

Of importance for clinical translation, a hoped-for benefit from an amplification of CD8^+^ T cell numbers by establishing CTLLs was confounded by a ~100-fold loss of activity/cell compared to ex vivo cell-sorted CD8^+^ T cells specific for the very same viral epitope and compared directly in the same experiment [77,78]. This shows an important advantage of the model over clinical trials where a controlled comparison by transferring cell culture-propagated CTLLs to one cohort of patients and ex vivo donor-derived purified CD8^+^ T cells to another cohort is unlikely to pass an ethics committee. These studies in the mouse model, thus, predicted that ex vivo isolated virus-specific CD8^+^ T cells are the best choice for an adoptive immunotherapy of CMV disease even though cell numbers available from donors are usually low.

The histological correlate of protection against tissue infection is the formation of nodular inflammatory foci (NIF) that represent micro-anatomical structures to which infection is confined by tissue-infiltrating antiviral CD8^+^ T cells selectively accumulating at infected tissue cells for delivering their effector functions. Importantly, the formation of protective NIF strictly depends on the presentation of the viral epitope on the infected tissue cells. This has been shown by transferring epitope-specific CD8^+^ T cells to recipient mice infected with a parental virus compared to recipient mice infected with a virus mutant for which the presentation of the respective protective viral epitope is precluded by a point mutation that replaces the usually hydrophobic C-terminal amino acid residue (Φ), which anchors an antigenic peptide to the presenting MHC molecule, with alanine (Ala; A) [78,79]. Clearly, such a stringent specificity control is not possible in clinical research.

**Figure 1:**
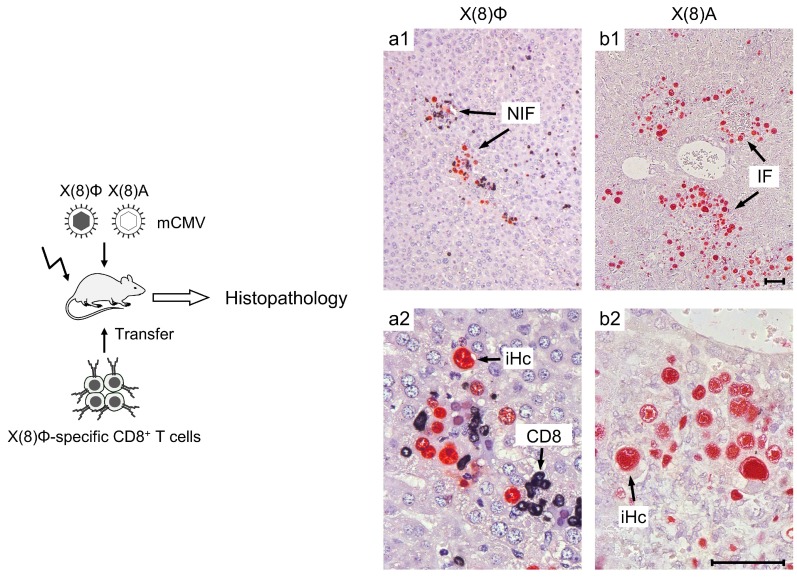
Basic model of immunotherapy of CMV disease. (Left) Experimental protocol. Mice representing designated HCT recipients become immunocompromised by hemato-ablative total-body γ- irradiation (flash symbol), which is followed by infection and by transfer of CD8^+^ T cells, either CTLLs or cells ex vivo isolated from infected, immunocompetent donor mice, which are both specific for the same viral antigenic peptide (mostly a nonapeptide) with the general sequence X(8)Φ, where X represents amino acid residues and Φ represents the MHC-anchoring C-terminal residue that is mostly a hydrophobic residue. For demonstrating epitope specificity of protection, mice become infected either with parental mCMV encoding the functional antigenic peptide X(8)Φ or with the virus mutant that encodes the nonfunctional sequence X(8)A. The mutation strategy provides dual security, since X(8)A mostly does not even exist as a peptide, because it is usually not generated in the proteasome in the first place. In addition, in case X(8)A is generated, it would fail to anchor to an MHC molecule. (Right) Representative examples of liver histopathology after CD8^+^ T cell transfer and infection with either parental virus mCMV-X(8)Φ (**a1**, overview, **a2**, detail) or mutant virus mCMV-X(8)A (**b1**, overview, **b2** detail). Bar markers represent 50 µm throughout. Immuno-histological staining identifies infected liver cells, which are primarily hepatocytes (iHC, red-stained) as well as tissue-infiltrating CD8^+^ T cells (CD8, black stained) NIF, nodular inflammatory focus. IF, focus of infection. The representative example is taken from Reference [79], PLOS Pathogens, 2015.

A point mutation is the minimal manipulation of viral genetics required to alter antigenicity and immunogenicity [30]. This strategy of deleting an epitope selectively is thus superior to the deletion of the entire peptide sequence or even more of the entire protein sequence. As the result, tissue cells infected with an epitope-encoding parental virus are attacked by the transferred CD8^+^ T cells within NIFs, which prevents viral intra-tissue spread and eventually resolves productive, cytopathogenic tissue infection. In contrast, the virus mutant spread unhindered within tissues, which is associated with viral histopathology visible as extensive foci of cytopathogenic infection and tissue destruction [78,79] (Figure 1).

The predictions from the BALB/c mouse model were largely confirmed by clinical trials. As a milestone in clinical immunotherapy of hCMV infection, seven years after the model’s prediction was published [43]. Riddell and colleagues reported successful restoration of immunity to hCMV in immuno-deficient humans by the adoptive transfer of cell culture-expanded hCMV-specific human CD8^+^ CTL clones [80]. Since then, numerous reports followed, refining CD8^+^ T cell immunotherapy in human HCT recipients that reactivate latent hCMV in consequence of the hematoablative anti-leukemia therapy. We apologize for not reviewing all progress made over the years in clinical immunotherapy but would like to emphasize the clinical verification of the important predictions made by the model discussed above. This includes the superior efficacy of viral epitope-specific CD8^+^ T cells isolated ex vivo from donors and transferred to HCT recipients with no preceding propagation in cell culture [81,82,83,84,85,86,87].

## 8. Challenging the Validity of the Model: CD8^+^ T Cells Are Dispensable for Controlling CMV

The model, as discussed above, has documented control of mCMV infection in the immunocompromised murine host by adoptive transfer of donor-derived virus-specific CD8^+^ T cells as well as by CD8^+^ T cells newly arising from T cell lineage progenitors in recipients of experimental HCT in the course of lympho-hematopoietic reconstitution. Moreover, CD4^+^ T cells plus non-T lymphocytes and accessory cells derived from the same donor mice or reconstituted in the same HCT recipients did not confer or mediate protection, which suggests that CD8^+^ T cells are the only antiviral effector cells that control the infection in the recipients. This conclusion was challenged by demonstrating a perfect control of mCMV infection in mice long-term depleted of CD8^+^ T cells (likely also of CD8^+^ DCs) by anti-CD8 antibodies prior to infection [88] as well as in mice genetically deficient in CD8^+^ T cell differentiation due to a knockout of the gene encoding β2-microglobulin that is essential for correct folding of the epitope-presenting pMHC-I complex [89]. These examples bring us back to our general considerations on clinical correlates discussed above. Clearly, experimental mouse mutants, while instrumental to basic research, rarely have a clinical correlateand, even if a respective genetic disorder existed in humans, the results would be casuistical, not applying to the majority of patients in routine clinical practice.

One has to bear in mind that elimination of cell types of the immune system alters the network of the immune cell and cytokine/chemokine crosstalk, which results in remodeling to a new immune system homeostasis in which cells of the reorganized immune system take roles that they do not take in the presence of the depleted cell type. Regarding reconstitution of the iatrogenically wiped-out immune system in the context of experimental HCT, a scenario that does have a clinical correlation in the form of clinical HCT, depletion of newly arising CD8^+^ T cells but not of CD4^+^ T cells results in disseminated multiple-organ mCMV infection including interstitial pneumonia with a lethal outcome [31,90]. For an explanation, we propose that in an experimental setting of infection occurring early after HCT, the time between cell depletion and disease manifestation is too short to remodel the immune system for educating alternative antiviral mechanisms on time.

Notably, despite CD8^+^ T cells being the physiological antiviral effector cell type during reconstitution after experimental HCT, diverse immune cell types including NK cells [91], memory B cells [92], CD4^+^ T cells [93], and γ/δ T cells [94,95] are capable of conferring protection when transferred as mature, differentiated effector cells not involving immune system remodeling. These findings from mouse models of immunotherapy by adoptive cell transfer are important, because they significantly broaden the spectrum of options for a clinical immunotherapy of CMV disease.

## 9. Challenging the Validity of the Model: Antigen-Specificity of Protective CD8^+^ T Cells

All CMVs express intranuclear immediate-early (IE) proteins under the control of a strong major IE (MIE) promoter-enhancer element [96] to kick-start the viral replication cycle by transactivating the subsequent kinetic class of viral genes, the early (E) genes [97]. Analysis of the antigen-specificity of protective antiviral CD8^+^ T cells in the BALB/c mouse model revealed a high frequency of CTL present in draining lymph nodes and recognizing infected target cells that were metabolically arrested in the IE phase [98,99]. At the time of this finding, antigen processing was still in its infancy and highly debated. Therefore, proteins localizing to the cell nucleus were then not considered to be antigens for the recognition by T cells. This is now science history and a nonapeptide, referred to as the IE1 peptide, derived from the mCMV IE1 protein by proteasomal processing and presented at the cell surface in complex with the murine MHC class-I molecule L^d^, was identified as the antigen recognized [100,101]. IE1-epitope specific CD8^+^ T cells, short-term CTLLs as well as ex vivoisolated memory CD8^+^ T cells, protected against lethal mCMV infection in the adoptive cell transfer model [77,102]. Accordingly, a model “vaccine” virus, specifically a recombinant vaccinia virus expressing the mCMV IE1 peptide integrated into the hepatitis B virus core antigen as a carrier protein, protected against a lethal high-dose challenge infection with mCMV [103]. IE1 specific CD8^+^ T cells were found to control mCMV latency in the lungs [104]. In infected BALB/c recipients of experimental HCT, IE1-specific effector-memory CD8^+^ T cells were found to accumulate over time in the latently infected lungs [105], which was the first description of a phenomenon now known as “memory inflation” and still under intense investigation [54,106,107,108].

Originally, the relevance of IE proteins in immunity to hCMV was denied and the mouse model was considered to be non-predictive in this particular aspect. Doubt was based on the finding that by far most hCMV-specific CD8^+^ T cells, which were derived from latently infected but otherwise healthy volunteers, recognized the hCMV antigen ppUL83/pp65. As the main component of the virion tegument, this protein is released into cells during the entry process and, thus, does not depend on viral gene expression for antigen processing and presentation to CD8^+^ T cells ([109,110], reviewed with comments on the mouse model in Reference [111]). The mouse model does not fundamentally differ in this respect, since early studies on the immune response to mCMV also revealed a strong CD8^+^ T cell response to virion antigens [98,99].

Years later, the mouse model was eventually rehabilitated by the technical advance of quantitating epitope-specific CD8^+^ T cells directly ex vivo, that is with no preceding expansion in cell culture. Techniques include the cytofluorometric detection of intracellular cytokines induced in CD8^+^ T cells upon short-term stimulation with antigenic peptides as well as detection of fluorochromated MHC/HLA-peptide multimers that bind to the respective TCRs. These new approaches revealed that, on the population level, the MIE locus is a hot spot for antigenicity also in humans [112,113,114], and a report soon followed to suggest a major role for hCMV IE1-specific CD8^+^ T cells in the protection against CMV disease in SOT, specifically in heart and lung transplantation recipients [115].

What actually has led to discrediting the murine model? Early studies on the antigen specificity of mCMV-specific CD8^+^ T cells were performed by isolating in vivo-sensitized CD8^+^ T cells from draining lymph nodes, followed by clonal expansion in limiting dilution cell cultures with recombinant interleukin 2 (rIL2) without adding virus or viral antigens [42]. Testing the specificity of derived CD8^+^ CTL clones on infected target cells arrested in stages of the viral gene expression cascade (see above) revealed a high frequency of IE-specific cells among CD8^+^ T cells activated in vivo to express IL2-receptors [98,99]. In contrast, CD8^+^ T cells specific for hCMV were usually derived from peripheral bloodby stimulation in cell culture with virion preparations or with APCs exposed to virion preparations of hCMV laboratory strains, mostly AD169. Such preparations are rich in non-infectious subviral particles, the dense bodies (DBs), which essentially represent enveloped viral tegument containing ppUL83/pp65 as a major constituent [116]. This causes a bias by an in vitro selection to the favor CTL clones specific for ppUL83/pp65 epitopes.

This example is a wholesome lesson about how a supposed nonconformance between a model and clinical investigation, discrediting the model, may simply be caused by a difference in methodology. Nonetheless, it is clear that, on the molecular level, antigenic peptides of hCMV presented by human MHC/HLA molecules cannot be predicted by any animal model. Accordingly, based on MHC/HLA polymorphism, different mouse strains select different viral peptides as epitopes for presentation (for lists of currently known antigenic peptides of mCMV, see References [71,117]) and, in the human population, each individual has her/his “private” viral antigenicity fingerprint depending on HLA type and likely also on the hCMV variants harbored [114].

## 10. Immune Evasion of CMVs Correctly Predicted by mCMV

CMVs all express immune evasion proteins interfering with recognition of infected cells by literally any subset of immune cells involved in innate or adaptive immunity. When an immune cell type is newly described, for instance innate lymphoid cells (ILCs) [118], and is found to play little or no role in controlling CMV infection, this almost definitely indicates that this cell type has fundamentally contributed to viral evolution during virus-host co-speciation by positive selection for virus variants that have evolved an immune evasion mechanism [119]. Accordingly, “negative” data call for an identification of the responsible immune evasion gene. We refrain here from reviewing the broad field of immune evasion, which is a topic on its own right. However, when discussing the validity of the murine model, we would at least like to recall that immune evasion of CD8^+^ T cells by CMV proteins that interfere with the MHC-I pathway of antigen processing and presentation were first described in the mouse model with the m152 gene product representing the prototype (reviewed in Reference [101]). Notably, the m152 protein simultaneously interferes with recognition by CD8^+^ T cells and NK cells by down modulating pMHC-I and RAE-1 family ligands of NK cell receptor NKG2D, respectively. As with many questions in CMV research, the most valuable contribution made by the studies on m152-mediated immune evasion in the mouse model was to have provided the first “proof of concept” for an in vivo relevance [50,120,121,122].

Interference of viral proteins with host immunity requires a very precise host adaptation reflected by “private” genes that have no homologs in CMVs of any other host species. Therefore, immune evasion genes of mCMV cannot predict corresponding genes of hCMV. What the model has revealed is the principle, even though biological convergence CMVs of different host species have evolved different genes and associated molecular modes of action to arrive at the same goal, namely to dampen immune control.

## 11. Results from the Mouse Model that Await Clinical Investigation

### 11.1. Verifying the Cellular Site of Latent Infection

Humanized mouse models (see Section 6) have already proven instrumental for providing experimental “proof of concept” for observational findings from clinical investigations. A prominent example that was not predictive but confirmative is the in vivo demonstration of hCMV latency and reactivation in hematopoietic cells [123]. In this study, hCD34^+^ hematopoietic stem cell (HSC)-engrafted NOD-scid IL2Rγc^null^ mice were acutely infected with hCMV by the administration of infected fibroblasts and then established a latent infection in monocytes/macrophages that migrated into organ tissues. Virus reactivation was triggered by granulocyte colony-stimulating factor, G-CSF. A hematopoietic-lineage site of latency was not reproducible for mCMV in a conventional mouse model [124] in which the latent virus could not be transferred to recipients with bone marrow cells from latently infected donors. Accordingly, reactivation leading to primary infection of the immunocompromised recipients was not detectable. This could be an example for a nonconformance between hCMV and mCMV latency, even though one must note that reactivation of hCMV from HSC of latently infected human donors remains to be shown in the humanized mouse model. In a conventional mouse model, liver sinusoidal lining cells (LSECs) were identified as one cellular site of mCMV latency [34].

### 11.2. Intravital Imaging: An Advantage of the Mouse Model

Intravital imaging using two-photon microscopy is a technique that allows an online study of the dynamics of immune cell interactions with infected cells in host tissues. This was recently used to study the formation of the previously known nodular inflammatory foci (NIF, see Figure 1) [125], which are the micro-anatomical structures in which antiviral effector cells attack infected tissue cells to confine and eventually clear productive infection in a viral epitope-specific manner [78]. A novel aspect provided by this approach in mCMV models is the conclusion that the in vivo killing capacity of cytolytic CD8^+^ T cells is more limited than previously assumed and depends on T cell cooperativity involving CD4^+^ T cells [126,127].

### 11.3. Role for Mast Cells in Controlling CMV Infection

Mast cells (MCs) were only recently considered as a player in the control of CMV infections. Using the mouse model, MCs were identified as a new cell type that is permissive to a CMV infection in vivo [128]. In addition, chemokine CCL5 secreted by virally-activated MCs was shown to more efficiently recruit antiviral CD8^+^ T cells to the lungs, which contributes to a faster resolution of a productive infection of the lungs [129]. Experiments are in progress to evaluate the importance of MC reconstitution in the model of mCMV infection of experimental HCT recipients for predicting a role for MC reconstitution in controlling hCMV infection in clinical HCT.

### 11.4. Role for Alternative Viral gH/gL Entry Complexes in the Initial Host Organ Colonization and Intra-Tissue Spread

The function of alternative host cell entry complexes in the hCMV envelope, namely the trimeric complex gH/gL/gO and the pentameric complex gH/gL/UL128-131A, are a topic in current hCMV research but are difficult to investigate in the human host. Likewise, mCMV expresses alternative entry complexes gH/gL/gO and gH/gL/MCK-2, putatively representing functional analogs of their hCMV counterparts. With a focus on liver infection, a recent study in the mouse model showed that gO of mCMV is critical for efficient organ entry of virus disseminated via the blood stream, regardless of entry cell type, that is the liver macrophage, the sinusoidal endothelial cell, or directly the hepatocyte [27] that represents the main virus-producer in the infected liver [32]. However, subsequent intra-tissue spread, which is the most relevant condition for histopathology, occurred in the absence of gO as well as in the absence of MCK-2 but failed in combined absence of gO and MCK-2, which indicates redundancy in that the two entry complexes can substitute for each other in mediating intra-tissue virus spread that results in liver pathology. Studies on virus entry receptors have a great translational potential for therapeutic interventions to prevent viral spread by blocking these receptors.

### 11.5. Improved Anti-Viral Efficacy of CD8^+^ T Cells by “Cognate Help” from MHC-I Redirected CD4^+^ T Helper Cells

**Figure 2:**
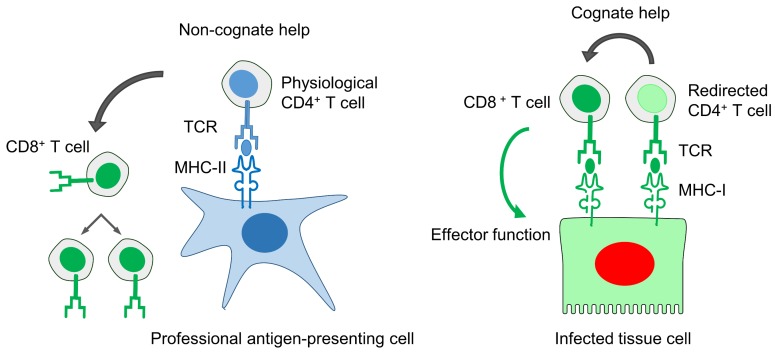
Models of “non-cognate” versus “cognate” help. See the body of the text for explanation. Blue: MHC/HLA-II-restricted. Green: MHC/HLA-I-restricted. Red: Infected. Reproduced, with modification, from Reference [130], *Medical Microbiology and Immunology*, Springer, 2016.

Early studies in the mouse model, using latently-infected and, thus, mCMV antigen-experienced mice as adoptive cell transfer donors, had unexpectedly revealed a missing impact of memory CD4^+^ T helper cells on the protective antiviral effect of transferred memory CD8^+^ T cells derived from the same donor mice [72]. It was then concluded that the memory CD8^+^ T cells neither need T cell help nor do they profit from the co-transfer of T helper cells in terms of instant protective function. To avoid a frequent misunderstanding, clinical data indicate a supportive role for T cell help in longer-term maintenance of the antiviral CD8^+^ T cell response (discussed in Reference [79]). However, based on our knowledge, a benefit from co-transfer of both T cell subsets for an instant control of acute infection has not yet been tested in clinical trials. A recent study in mice “humanized” for antigen presentation [79], specifically HLA-A*02 (human MHC-I)-transgenic mice infected with a recombinant mCMV coding for a dominant antigenic peptide of hCMV protein ppUL83/pp65, a peptide that is presented by the HLA-A*02 molecule, gives a clue for understanding the “missing help paradox.” In this work, protection against the antigenically humanized recombinant virus by transfer of human CD8^+^ T cells transduced with a TCR specific for the HLA-A*02-ppUL83 peptide complex was improved by co-transfer of CD4^+^ T cells transduced with the same MHC-I-restricted TCR. This finding led us to propose the model of “cognate help” delivered in close proximity as opposed to “non-cognate” help delivered systemically over a distance [130]. While all infected host tissue cells, such as stromal and parenchymal cells involved in viral histopathology, express MHC-I molecules, most cell types are constitutively negative or at best inducibly positive for MHC-II antigens. As a consequence, physiological CD4^+^ helper T cells, which are MHC-II-restricted, cannot bind to every infected cell type to which an antiviral CD8^+^ effector cell binds and, therefore, cannot deliver helper cytokines directly next to the effector cell. In contrast, direct help can be provided in close proximity to the CD8^+^ effector T cell by a CD4^+^ T cell redirected to epitope-presenting MHC-I molecules by an MHC-I-restricted, engineered TCR (Figure 2). Clearly, this new understanding of T cell help is important for improving the immunotherapy of clinical CMV infection.

### 11.6. Preventing Graft-versus-Host Disease in Allogeneic HCT by Regulatory T Cells

In clinical HCT, transplantation donor and recipient are usually MHC/HLA matched but differ in minor transplantation antigens, which defines allogeneic HCT and bears a risk of GvH reaction and associated disease. Mouse models of allogeneic HCT and mCMV infection, however, usually enforce GvHD by MHC disparity or even by infusion of mature T cells [131]. Recent work showed an in vivo expansion of recipient regulatory T cells before MHC-disparate allogeneic HCT reduces the risk of GvHD without preventing the reconstitution of antiviral CD8^+^ effector T cells [132]. These findings are promising in that deleterious GvHD may be prevented in allogeneic HCT without preventing the beneficial graft-versus-infection (GvI) response.

## 12. Conclusions

With focus on the mouse model of CMV infection after experimental HCT, a model in which viral pathogenesis and reconstitution of protective immunity are interdependent [133], we hope to have sharpened the senses regarding the importance of proper model design for generating valid models of predictive value for clinical investigation. The first step in model design must be to identify and reproduce the medical cornerstone facts defined by the clinical correlate. In awareness of the impossibility to precisely reproduce human disease in any animal model, not even in NHP models, questions asked to models can reveal principles but never a complete fit in details. The mouse model of CMV infection has elucidated important principles that have survived the “test of time” and that went into clinical translation.

## Figures and Tables

**Table 1:** Strengths and weaknesses of conventional and humanized mouse models.

Parameter	Conventional Mouse Model	Humanized Mouse Model
Human virus	No	Yes
Human host	No	Chimeric
Virus-host adaptation	Yes	Partial
Ethical concerns	Moderate	Donor consent required
Technical demands	Low	High
Statistical demands	Easy to fulfill	Difficult to fulfill
Morbidity-mortality studies	Yes	No
Comprehensive organ disease	Yes	No
Viral histopathology	Yes	Restricted to implants
Intra-host virus spread	Yes	Limited
Host-to-host transmission	Yes	No
Cytokine signaling	Intact	Partially disturbed
Host genetic variance	Yes, strains or targeted mutation	Limited to donor typing
Virus genetic variance	Yes, isolates or targeted mutation	Yes, isolates or targeted mutation
Co- and super-infection	Yes	Yes
Test and control cohort identity	Yes	Limited by donor material
Immunotherapy	Yes	Restricted to implants
Testing of antivirals	Doubtful	Yes, though with caution
Intravital imaging	Yes	Yes
Model for fetal brain infection	Yes	Unrealistic
